# Effects of Dietary Selenium Deficiency or Excess on Selenoprotein Gene Expression in the Spleen Tissue of Pigs

**DOI:** 10.3390/ani9121122

**Published:** 2019-12-11

**Authors:** Zhuang Lu, Pengzu Wang, Teng Teng, Baoming Shi, Anshan Shan, Xin Gen Lei

**Affiliations:** 1 Institute of Animal Nutrition, Northeast Agricultural University, Harbin 150030, China; luzhuang0416@163.com (Z.L.); princewpz@163.com (P.W.); tt645742243@163.com (T.T.); asshan@neau.edu.cn (A.S.); 2 Department of Animal Science, Cornell University, Ithaca, NY 14853, USA; xl20@cornell.edu

**Keywords:** pig, spleen, selenium, selenoprotein

## Abstract

**Simple Summary:**

Selenium (Se) is an essential nutrient for humans and many other species including pigs, and it plays an important role in many aspects of biological functions especially in the immune system. The metabolic roles of Se implement its biological function through its incorporation into selenoproteins, that contain a unique amino acid selenocysteine (Sec). Although 24–25 selenoprotein genes have been identified in mammals as the most important immune organ, the expression pattern of selenoproteins in the spleen of pigs regulated by different Se levels remains poorly known. The present study is conducted to evaluate the effects of dietary Se deficiency and excess on the mRNA expression levels of selenoproteins in pig spleen tissues. The results show that dietary Se levels can significantly affect the transcription levels of 14 selenoprotein genes in the spleen of pigs, which can help us to understand the biological functions of Se further and improve the use of Se in livestock production.

**Abstract:**

To evaluate the effects of dietary Se deficiency and excess on the mRNA levels of selenoproteins in pig spleen tissues, 20 healthy uncastrated boars (Duroc × Landrace × Yorkshire, 10 ± 0.72 kg) were randomly divided into four groups (5 pigs per group). The pigs were fed a Se deficient corn-soybean basal feed (Se content <0.03 mg/kg) or basal feed with added sodium selenite at 0.3, 1.0, or 3.0 mg Se/kg diet, respectively. The experiment lasted 16 weeks. The spleen tissue was collected to examine the mRNA expression levels of 24 selenoprotein genes at the end of the study. Compared with pigs in other groups, those fed with the 1.0 mg Se/kg diet had higher mRNA levels of *glutathione peroxidase 1* (*Gpx1*), *glutathione peroxidase 2* (*Gpx2*), *deiodinase type II* (*Dio2*), *thioredoxin reductase 3* (*Txnrd3*), *selenoprotein H* (*Selh*), *selenoprotein N, 1* (*Sepn1*), *selenoprotein P1* (*Sepp1*), and *selenoprotein V* (*Selv*) in the spleen (*p* < 0.05). Dietary Se deficiency resulted in lower mRNA levels of *Gpx1*, *Gpx2*, *glutathione peroxidase 3* (*Gpx3*), *Dio2*, *thioredoxin reductase 2* (*Txnrd2*), *Txnrd3*, *Selh*, *selenoprotein* *I* (*Seli*), *selenoprotein K* (*Selk*), *selenoprotein M* (*Selm*), *Sepn1*, *Sepp1*, and *Selv* in the spleen than the other three groups. Dietary Se levels did not affect the mRNA levels of *glutathione peroxidase 4* (*Gpx4*), *deiodinase type* I (*Dio1*), *deiodinase type* III (*Dio*3), *selenophosphate synthetase 2* (*Sephs2*), *thioredoxin reductase 1* (*Txnrd1*), *selenoprotein O* (*Selo*), *selenoprotein S* (*Sels*), *selenoprotein W* (*Selw*), *selenoprotein X* (*Selx*), and *selenoprotein 15* (*Sel15*) in the spleen (*p* > 0.05). Dietary Se levels can affect the transcription levels of 14 selenoprotein genes in the spleen of pigs.

## 1. Introduction

Selenium (Se) is a nutritionally essential trace element for humans and many other species [1]. Previous data have suggested that Se plays an important role in chemopreventive effects [2,3], muscle metabolism [4,5], oxidant defense [6], neurobiology [7], aging [8], and reproduction [9]. Many previous studies reported that Se also plays an important role in the immune system [10,11]. In a mitogen-stimulated situation, Se deficiency suppresses lymphocyte proliferation, reduces the level of immunoglobulin, and declines the number of T cells [12,13]. However, excess dietary Se can also damage the normal function of the immune system [14,15,16,17]. It is found that excess dietary Se caused lymphocytic necrosis [14], growth retardation, and atrophy of immune organs [18].

Composing Se-containing proteins is the main method that Se exhibits its biological functions [19,20]. Therefore, the levels of Se in the feed have an important influence on the levels of selenoproteins. There are 24–25 selenoprotein genes identified in mammals, but the effects of dietary Se concentrations ranging from deficiency to moderately high levels on the gene expression of selenoproteins are still unclear [21,22,23]. Selenoproteins are essential for the immune system, and knockout of all T-cell selenoproteins can induce a decrease in the number of T cells and atrophy of many immune organs [24]. The spleen is the most important immune organ. Thus, it is crucial to investigate the relation between the profile of selenoprotein gene expression and the metabolic impact of dietary Se deficiency or excess in the spleen. Because pigs are not only an important kind of food-producing animal but also an excellent model for human nutrition [25,26] and medicine [27,28], revealing the role of dietary Se in the regulation of selenogenome expression in pigs will help understand Se metabolic diseases of animals, as well provide useful clues for human health. Therefore, the present study is conducted to evaluate the effects of dietary Se deficiency and excess on the mRNA expression levels of selenoproteins in pig spleen tissues.

## 2. Materials and Methods

### 2.1. Ethical Approval

All procedures used in this study were approved by the Institutional Animal Care and Use Committee of Northeast Agricultural University.

### 2.2. Pigs and Diets

A total of 20 healthy uncastrated boars (Duroc × Landrace × Yorkshire) with an initial body weight of 10 ± 0.72 kg were randomly divided into four groups (5 pigs per group). The pigs were fed a Se deficient corn-soybean basal diet (BD, produced in the Se deficient area in Heilongjiang, China, Se content <0.03 mg/kg diet), or the BD added sodium selenite at 0.3, 1.0, or 3.0 mg Se/kg diet, respectively. The experiment lasted 16 weeks. All pigs were located in individual cages with fully slatted flooring, a single-hole feeder, and a nipple waterer, and the feed and water were available ad libitum. At the end of the study, all pigs were slaughtered. The spleen samples were quickly removed, rinsed with ice-cold sterile deionized water, frozen immediately in liquid nitrogen, and stored at −80 °C until required.

### 2.3. Quantification of Selenoproteins mRNA

Total RNA was isolated from the spleen tissue (50 mg tissue; n = 5/diet group) using the Trizol reagent according to the manufacturer’s instructions (Invitrogen, Shanghai, China). The concentration and quality of the RNA were determined by an ultramicrospectrophotometer (Thermo Scientific, Wilmington, DE, USA) and agarose-gel electrophoresis, respectively. The cDNA was generated from total RNA using a cDNA reverse transcription kit (Takara, Dalian, China) and stored at −80 °C before use.

The expression levels of selenoprotein genes were determined by the technology of real-time quantitative reverse transcription PCR using SYBR Premix ExTaq TM (Takara, Dalian, China) on the ABI PRISM 7500 system (Applied Biosystems, Forster City, CA, USA). The PCR primers (Table 1) were designed and synthesized by Sangon (Shanghai, China). The reaction volume was 20 μL, as recommended by the SYBR real-time PCR kit (Takara, Dalian, China). The RT-PCR conditions were as follows: 1 cycle at 95 °C for 30 s, 40 cycles at 95 °C for 5 s, and 60 °C for 34 s. All of the PCR reactions were performed in triplicate, and the results were normalized on the basis of β-actin gene expression. Relative mRNA expressions were calculated using the 2^−ΔΔCt^ method [29].

### 2.4. Statistical Analysis

One-way ANOVA followed by Duncan’s multiple range test (SPSS for Windows 13.0, Chicago, IL, USA) was used to test the effects of the four dietary Se concentrations on mRNA levels of selenoproteins. The data were expressed as the mean ± standard deviation. The differences were considered to be significant at *p* < 0.05.

## 3. Results

**Figure 1:**
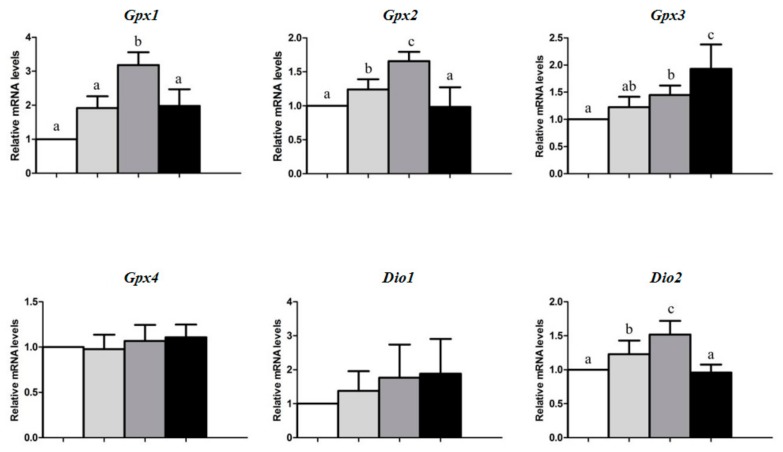

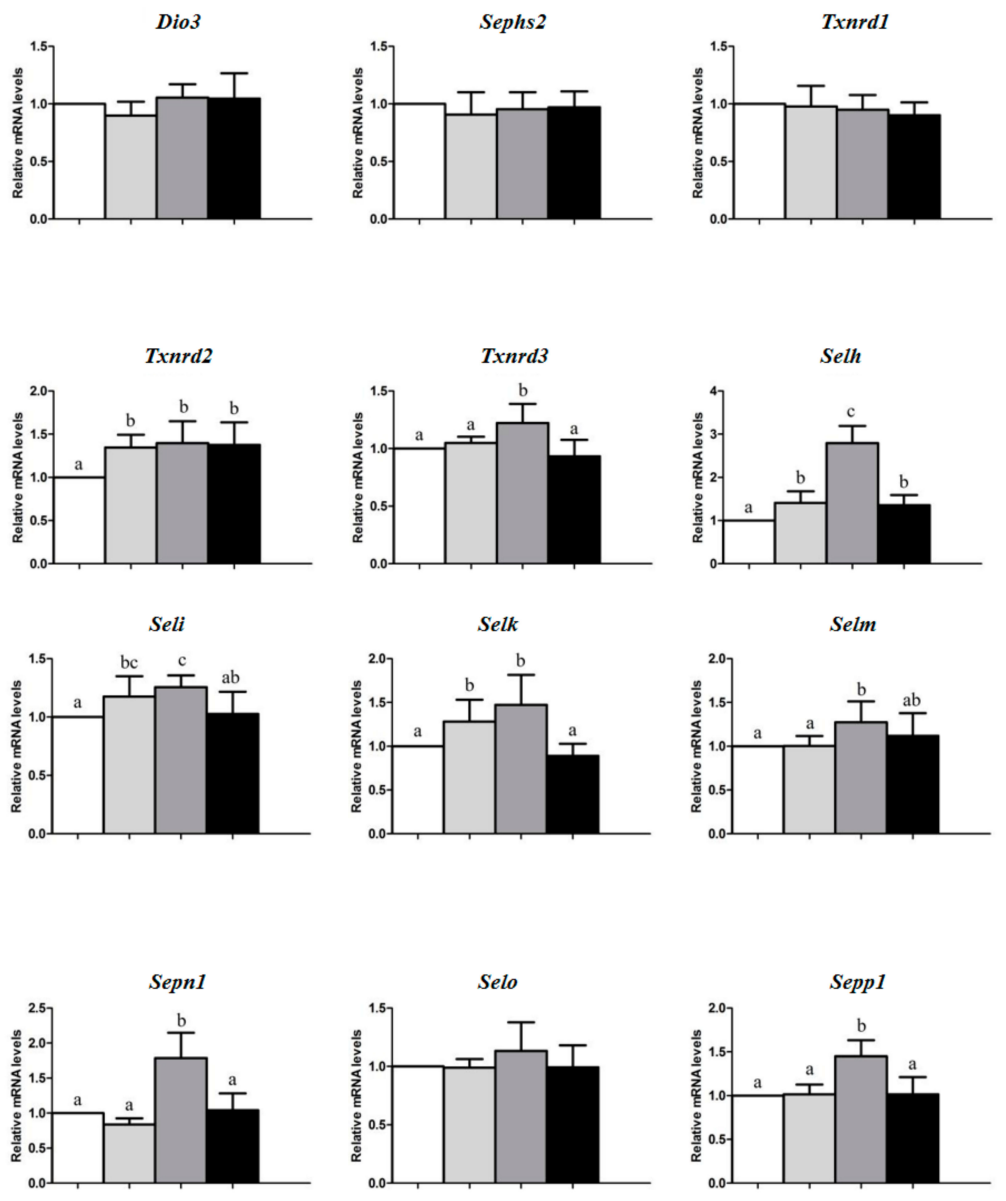

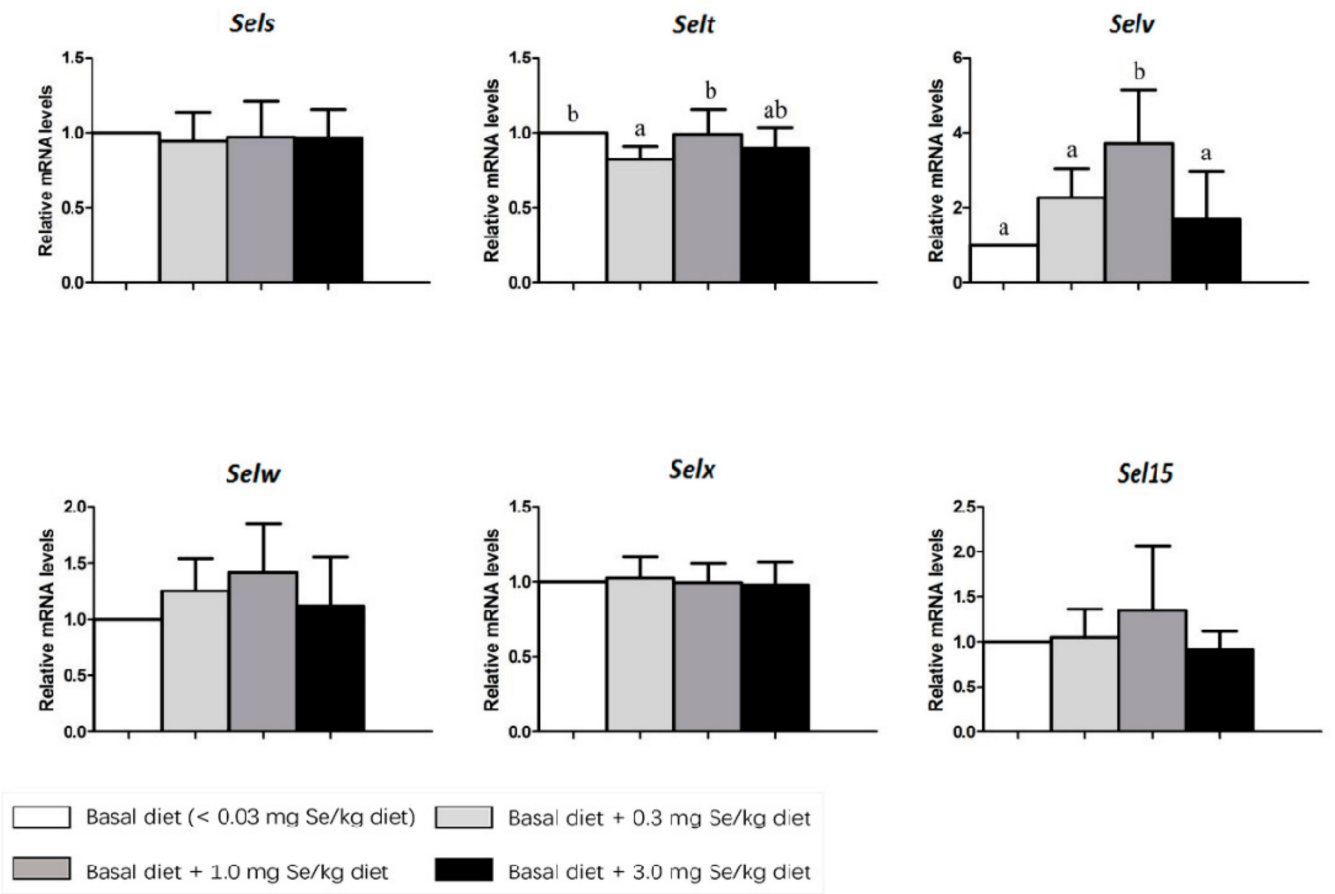
Effects of dietary Se concentrations on mRNA levels of selenoprotein genes in the spleen of pigs at wk 16. Data are means ± SDs, n = 5. Means stands for a gene without a common letter differ, *p* < 0.05. *Gpx: glutathione peroxidase; Dio: deiodinase type*; *Sephs: Selenophosphate synthetase; Txnrd: Thioredoxin reductase; Selh: Selenoprotein H; Seli: Selenoprotein I; Selk: Selenoprotein K; Selm: Selenoprotein M; Sepn1: Selenoprotein N, 1; Selo: Selenoprotein O; Sepp1: Selenoprotein P1; Sels: Selenoprotein S; Selt: Selenoprotein T; Selv: Selenoprotein V; Selw: Selenoprotein W; Selx: Selenoprotein X; Sep15: Selenoprotein 15.*

Compared with pigs in other groups, as shown in Figure 1, those fed with the 1.0 mg Se/kg diet had higher (*p* < 0.05) mRNA levels of *Gpx1*, *Gpx2*, *Dio2*, *Txnrd3*, *Selh*, *Sepn1*, *Sepp1*, and *Selv* in the spleen. However, pigs fed 3.0 mg Se/kg diet had the highest (*p* < 0.05) mRNA levels of *Gpx3* in the spleen.

Dietary Se deficiency resulted in lower mRNA levels of *Gpx1*, *Gpx2*, *Gpx3*, *Dio2*, *Txnrd2*, *Txnrd3*, *Selh*, *Seli*, *Selk*, *Selm*, *Sepn1*, *Sepp1*, and *Selv* in the spleen than the other three groups. Pigs fed 0.3 mg Se/kg diet had lower (*p* < 0.05) mRNA levels of *Selt* in the spleen than those fed with BD and 1.0 mg Se/kg diet.

Dietary Se levels did not affect the mRNA levels of *Gpx4*, *Dio1*, *Dio3*, *Sephs2*, *Txnrd1*, *Selo*, *Sels*, *Selw*, *Selx*, and *Sel15* in the spleen (*p* > 0.05).

## 4. Discussion

Both excess and deficiency of Se supply led to impaired health. Many previous studies showed that Se exhibits its biological functions by composing Se-containing proteins, especially in immune organs [24,30]. To date, scientists have been successful in describing in details the biological functions of many of the 24 selenoproteins studied, but the functions of others, such as Seli, Selk, Selo, remain unknown [31]. GPx is the first identified selenoprotein and mainly in the liver. The GPx family including GPxl, GPx2, GPx3, and GPx4, are involved in the catabolism of peroxides [32]. In this study, the results showed that the abundance of *Gpx1* and *Gpx2* in the 1.0 mg Se/kg diet group were significantly higher than those in other groups. Similarly, in a previous study, Gpx1 activity in the liver of pigs was elevated by increasing dietary Se supplementation from 0 to 3.0 mg/kg [23]. Interestingly, some studies indicated that Gpx1 activity can’t be enhanced by increasing dietary Se supplementation from 0.1 to 0.3 mg/kg in thyroid and pituitary of pigs [32]. The difference may be from the different dietary Se levels, treatment time, and organ species. The mRNA expression level of *Gpx3* was increasing with the dietary Se intake. This trend is consistent with the result of the previous study, showing the effect of Se on Gpx3 expression in pig testis tissues [23]. However, the dietary Se level did not affect the mRNA expression levels of *Gpx4*, which is similar to the results of a previous study in rats [33]. The selenoproteins in the Gpx family play an important role in the antioxidative system of animals, and the redox balance is closely related to immune function [34].

Iodothyronine deiodinases (Dio) are also an important group of selenoenzymes. The Dio family consists of three enzymes: types 1, 2, and 3 (D1, D2, and D3; or Dio1, Dio2, and Dio3). In humans, thyroid hormone metabolism can be influenced by dietary Se levels. Thyroid hormone can convert T4 prohormone to T3, and this conversion is performed by Dio1 or Dio2 [35]. The selenoproteins in the Dio family are also sensitive to Se levels, and all of them are reduced in the chicken thyroid in Se-deficient conditions [36]. In the present study, dietary Se did not affect the mRNA expression levels of *Dio1* or *Dio3* in spleens. However, several studies have shown that the mRNA expression of *Dio1* in the liver and kidney of mice can be significantly decreased by Se deficiency [37], and the discrepancy may be from the different species and organs. A significant increase in *Dio2* mRNA levels was observed in the spleen tissues of pigs fed diets containing Se of 0.3 and 1.0 mg/kg, while the *Dio2* mRNA levels decreased in the pigs fed the diet containing Se of 3.0 mg/kg. Similarly, Arthur et al. found that the enzyme activity of *Dio2* decreased in the brown adipose tissues of Se-deficient rats [38].

Selenophosphate synthetase 2 (SPS2) is an enzyme involved in the biosynthesis of selenoproteins. A study by Wang et al. noted that SPS2 itself was a selenoprotein, which could adjust on its own when synthesizing itself, and it could also adjust the synthesis of other selenoproteins [39]. In the present study, there was no significant difference between the groups of the mRNA levels of *SPS2*. In a previous study, similar changes were found in multiple organs of pigs [40].

Thioredoxin reductase (Txnrd) catalyzes the NADPH dependent reduction of thioredoxin and therefore plays a regulatory role in its metabolic activity [41]. There are three mammalian Txnrds. Their functions are antioxidant defense, redox regulation, and cell signaling [31]. Increasing Se intake can enhance the protein expression of Txnrd1 and Txnrd2 [42]. In the present study, the gene expression of *TrxR2* was increased in pig spleens by Se supplement, but the gene expression of *TrxR1* was unchanged. Besides, the gene expression of *TrxR3* was increased in the pigs fed the diet containing Se of 1.0 mg/kg, which is similar to the results of a previous study in chickens [43].

Selh is widely distributed throughout a variety of tissues and is higher in the early stages of embryonic development [44]. The gene expression of *Selh* is highly sensitive to Se intake [37]. In the present study, the gene expression of *Selh* is significantly reduced by a Se-deficiency diet. The decreased expression of *Selh* was related to the lack of Se in the spleen tissues. Se is the necessary material to synthesize Selh, and its deficiency results in a significant decrease in the expression of Selh. A recent study found that Seli contains the sequence homology to enzymes involved in the phospholipid synthesis. In the present study, the expression levels of *Seli* increased with the dietary Se intake and reached a peak at the Se concentration of 1.0 mg/kg, and then declined at a 3.0 mg/kg diet. In a previous study, similar changes were found in adipose tissues of chickens [23]. Selk, Sels, Seln, Sel15, and Selm are endoplasmic reticulum (ER) proteins that appear to be involved in redox balance and the unfolded protein response [31,45,46]. The greatest increases in *Selk*, *Selm*, and *Seln* mRNA expression were observed in the spleen tissue of pigs fed the diet containing a 1.0 mg Se/kg diet. This trend is consistent with the result of Liu’s study, showing the effects of Se on Selm expression in the same tissue [23]. These data suggest that the transcription of *Selk*, *SelM*, and *SelN* genes in the spleen tissues of pigs are more sensitive to Se. On the contrary, there were no significant differences in the mRNA expression levels of *Sels* and *Sel15* among the four groups. Approximately 60% of Se in plasma is incorporated in Selp, which contains 10 Se atoms per molecule as selenocysteine. In the present study, the mRNA levels of *Sepp1* were significantly increased in the 1.0 mg Se/kg diet group. Numerous studies confirm that Selp is closely related to the Se level of animals. It has been recently identified as a member of the redoxin protein family-like Selt, Selw, and Selv (also including Selh), based on the occurrence in its primary structure of a “thioredoxin-like fold” containing a selenocysteine [31]. In the present study, dietary Se did not affect the mRNA levels of *Selw* in spleens. This is consistent with the result of Yu’s study [47]. Interestingly, the lowest mRNA expression of *Selt* was observed in the spleen tissue of pigs fed the diet containing 0.3 mg Se/kg diet. In a previous study, similar changes were found in the liver tissue of pigs [23]. The mRNA levels of *Selo* and *Selx* were not affected by dietary Se. The results suggested that these selenoproteins are not sensitive to Se intake.

## 5. Conclusions

In conclusion, under the conditions of dietary selenium levels in this study, 24 selenoprotein genes are expressed in the pig spleen. The transcription levels of 14 selenoprotein genes in the pig spleen were regulated by Se level in diet, but others cannot be affected by the Se level of diet. The mRNA expression of *Gpx1*, *Gpx2*, *Dio2*, *Txnrd3*, *Selh*, *Sepn1*, *Sepp1*, and *Selv* is higher in the spleen of the pigs with the 1.0 mg Se/kg diet. Thus, within the studied levels, 1.0 mg Se/kg diet is an optimum dose for the expression of selenoproteins in the spleen of the pigs. In addition, pigs may be a good model for studying mechanisms related to the potential risk of high-Se or low-Se intake in humans.

## Figures and Tables

**Table 1:** Gene-specific primers for 24 selenoproteins and *β-actin* used in realtime quantitative reverse transcription PCR.

Gene	Accession Number	Primer Sequence (5′→3′)
*Glutathione peroxidase 1 (Gpx1)*	AF532927	GATGCCACTGCCCTCATGA
		TCGAAGTTCCATGCGATGTC
*Glutathione peroxidase 2 (Gpx2)*	DQ898282	AGAATGTGGCCTCGCTCTGA
		GGCATTGCAGCTCGTTGAG
*Glutathione peroxidase 3 (Gpx3)*	AY368622	TGCACTGCAGGAAGAGTTTGAA
		CCGGTTCCTGTTTTCCAAATT
*Glutathione peroxidase 4 (Gpx4)*	NM_214407	TGAGGCAAGACGGAGGTAAACT
		TCCGTAAACCACACTCAGCATATC
*Deiodinase type I (Dio1)*	AY533206	CATGGCCAAGAACCCTCACT
		CCAGAAATACTGGGCACTGAAGA
*Deiodinase type II (Dio2)*	NM_001001626	GCCACTTGACCTCCTTTCACT
		CTGGTTCTGGTGCTTCTTCAC
*Deiodinase type III (Dio3)*	AY533208	TGAAGTGGAGCTCAACAGTGATG
		TGTCGTCAGACACGCAGATAGG
*Selenophosphate synthetase 2 (Sephs2)*	EF033624	TGGCTTGATGCACACGTTTAA
		TGCGAGTGTCCCAGAATGC
*Thioredoxin reductase 1 (Txnrd1)*	AF537300	GATTTAACAAGCGGGTCATGGT
		CAACCTACATTCACACACGTTCCT
*Thioredoxin reductase 2 (Txnrd2)*	NM_001168702	CAATGCTACGACCTCCTGGT
		GGCGAAGGGCTCACATAGTC
*Thioredoxin reductase 3 (Txnrd3)*	XM_005669829	CACTTTCAGCATCCACCACAT
		TAAATCCATCCCTTTCCTCGT
*Selenoprotein H (Selh)*	NM_001184948	GAAGGGAATGGAGGAGGCTA
		TTCACCCTCACTGGAAGCTC
*Selenoprotein I (Seli)*	NM_001244662	GATGGTGTGGATGGAAAGCAA
		GCCATGGTCAAAGAGTTCTCCTA
*Selenoprotein K (Selk)*	DQ372075	CAGGAAACCCCCCTAGAAGAA
		CTCATCCACCGGCCATTG
*Selenoprotein M (Selm)*	FJ968780	CAGCTGAATCGCCTCAAAGAG
		GAGATGTTTCATGACCAGGTTGTG
*Selenoprotein N, 1 (Sepn1)*	EF113595	ACCTGGTCCCTGGTGAAAGAG
		AGGCCAGCCAGCTTCTTGT
*Selenoprotein O (Selo)*	AK236851	CTTCCGACCCCAGATGGAT
		GGTTCGACTGTGCCAGCAT
*Selenoprotein P1 (Sepp1)*	EF113596	AACCAGAAGCGCCAGACACT
		TGCTGGCATATCTCAGTTCTCAGA
*Selenoprotein S (Sels)*	GU983865	ACAGGAGGCTTTAGCAGCAG
		CGCTGTCCCATCTTTCAATC
*Selenoprotein T (Selt)*	NM_001163408	CGCTGCTCAAATTCCAGATA
		CTCTCCTTCAATGCGGATGT
*Selenoprotein V (Selv)*	GQ478346	CACTGGTCGCCAATGGATTC
		AGTGGCCAACGGAGAAAGC
*Selenoprotein W (Selw)*	NM_213977	CACCCCTGTCTCCCTGCAT
		GAGCAGGATCACCCCAAACA
*Selenoprotein X (Selx_)*	EF113597	ATCCCTAAAGGCCAAGAATCATC
		GGCCACCAAGCAGTGTTCA
*Selenoprotein 15 (Sep15)*	EF178474	ACAGCCCTGCCAAGCAGAT
		AACAGGGAGGCTGGGTAACAC
*β-actin*	AY550069	CCCAAAGCCAACCGTGAGAA
		CCACGTACATGGCTGGGGTG
